# Raman spectroscopy reveals growth phase-dependent molecular differences in bacterial membrane vesicles

**DOI:** 10.1128/jb.00410-25

**Published:** 2025-12-05

**Authors:** Lennart Christe, Annika Haessler, Stefanie Gier, Bernd Schmeck, Nathalie Jung, Maike Windbergs

**Affiliations:** 1 Institute of Pharmaceutical Technology, Goethe University Frankfurt 9173, Frankfurt, Germany; 2 Institute for Lung Research, Philipps University Marburg 9377, Marburg, Germany; 3 German Center for Lung Research (DZL) 542891, Giessen, Germany; 4 German Center for Infectious Disease Research (DZIF) 459706, Brunswick, Germany; 5 Institute for Lung Health (ILH), Justus-Liebig University 9175, Giessen, Germany; 6 Department of Medicine, Pulmonary and Critical Care Medicine, University Hospital Giessen and Marburg, Philipps University Marburg 9377, Marburg, Germany; University of Southern, California, Los Angeles, USA

**Keywords:** *Pseudomonas aeruginosa*, bacterial vesicle, growth phases, Raman spectroscopy, BMV

## Abstract

Bacterial membrane vesicles (BMVs) have attracted significant attention as highly efficient transport vehicles for molecules crossing biological barriers and as key mediators in infection processes. Based on this increasing interest, the need for standardized isolation protocols and comprehensive analytical approaches becomes apparent. Here, we evaluated BMVs from the human pathogen *Pseudomonas aeruginosa*, isolated at six distinct growth phases, using physicochemical assays, functional characterization, and Raman spectroscopy. Conventional analyses revealed growth phase-dependent differences in protein content, surface charge, and immunogenicity. Raman spectroscopy provided detailed molecular fingerprints, identifying shifts in protein-to-lipid ratios, increased lipid saturation, and alterations in protein secondary structure during later growth phases. Importantly, the absence of nucleic acid-specific spectral markers confirmed the outer membrane origin of the vesicles. Together, these findings demonstrate that the timing of BMV isolation critically determines their molecular composition and functional properties and establish Raman spectroscopy as a powerful label-free tool for semi-quantitative profiling of BMVs.

**IMPORTANCE:**

*Pseudomonas aeruginosa* is an opportunistic gram-negative pathogen and a leading cause of severe nosocomial infections. Its secreted bacterial membrane vesicles (BMVs) are increasingly recognized as mediators of pathogenicity and as potential therapeutic delivery systems. However, the lack of standardized and sensitive analytical techniques has hindered systematic characterization. Our study highlights the profound impact of the bacterial growth phase on BMV composition and immunogenicity. It introduces Raman spectroscopy as a chemically selective, label-free method for detecting subtle yet biologically relevant molecular changes. These insights provide a framework for improved standardization in BMV research and underscore the potential of Raman-based approaches in advancing both fundamental microbiology and translational applications.

## INTRODUCTION

Vesicular transport systems are indispensable to all forms of life, fulfilling critical tasks in cellular communication and environmental interactions (1). Vesicles typically consist of a lipid bilayer, effectively shielding enclosed molecules, such as fragile and enzymatic degradation-prone nucleic acids and proteins, and allowing their transport (2, 3). These extraordinary features of vesicles make them an attractive focus for research, potentially serving as a natural model for the development of biomimetic drug delivery systems, which are already being explored and applied (3–5).

In prokaryotes, vesicles are vital not only for nutrient uptake and signal transduction but also for different defense mechanisms (2). Hence, virulence factors or antibiotic resistance-mediating enzymes are packaged within bacterial membrane vesicles (BMVs), which bud off the bacterial membrane (6–8). The opportunistic bacterium *Pseudomonas aeruginosa*, classified as one of the highly problematic ESKAPE pathogens, is particularly adept at transferring virulence factors during infection using BMVs, often causing severe pneumonia (9, 10). Therefore, vesicles originating from *P. aeruginosa* are particularly intriguing for further investigation.

As a gram-negative bacterium, *P. aeruginosa* is enveloped by an inner and outer cell membrane separated by a peptidoglycan cell wall, and its secreted vesicles are classified according to their respective budding origin (11). Vesicles formed through outward bulging of the outer membrane are referred to as outer membrane vesicles (OMVs), while those involving both membranes are termed outer-inner membrane vesicles (12–14). Additionally, vesicular bodies can be formed by the lytic breakdown of cells (15, 16). A detailed classification of bacterial vesicles has been described in the literature, but for the sake of simplicity, all types will be referred to as BMVs throughout this text (2). Their chemical composition is influenced not only by vesicle biogenesis but also by the physiological state of the bacterium, which changes across the growth cycle. In many publications focusing on BMVs, the influence of these growth phases has received little attention, resulting in studies not specifying the exact time of isolation, consequently making it challenging to compare and reproduce BMV characteristics. Analytical challenges further impede the comparison: while standard approaches focus on fast, user-friendly physicochemical characterizations that omit discriminative chemical features of isolates, more advanced approaches like liquid chromatography-mass spectrometry (LC-MS) allow for in-depth characterization at the cost of labor-intensive sample preparation and destruction of the sample (17–20). Thus, new approaches for BMV characterization, such as Raman spectroscopy, which balances accessible experimental implementations with chemically discriminative analyses, are needed. This spectroscopic technique is based on the Raman effect, where light is partially inelastically scattered when interacting with matter. The resulting wavelengths of the scattered photons depend on energy changes in excited molecules, providing information about the chemical composition and molecular constitution (21). Raman spectroscopy, therefore, does not require extensive sample preparation, providing a label-free non-destructive analysis approach (22–25). These features position Raman analysis as a powerful tool in the field of bacteriology, e.g., for the diagnosis of bacterial infections in humans or tracking bacterial metabolism (26, 27). However, its use in BMV characterization remains limited (28, 29).

This study aimed to investigate the influence of bacterial growth phases on the BMV properties using planktonic *P. aeruginosa* cultures sampled at six different time points over 24 h. Established physicochemical characterization techniques, such as dynamic light scattering (DLS) and atomic force microscopy (AFM) and functional assays were combined with Raman spectroscopy to detect subtle chemical differences in BMV isolates and to offer a more comprehensive understanding of the molecular changes in BMVs depending on the isolation time point.

## MATERIALS AND METHODS

All experiments involving *P. aeruginosa* were conducted under biosafety level 2 containment conditions, in compliance with institutional guidelines for work with risk group 2 pathogens. No genetic modification of the used strain was performed.

### Bacteria cultivation

*P. aeruginosa* ATCC 27853 (Thermo Scientific, Waltham, USA) was streaked on Nutrient Broth agar plates (Oxoid Ltd., Basingstoke, UK) and incubated overnight at 37°C. A single colony was used to inoculate the pre-cultures (20 mL, grown in closed 50 mL polypropylene tubes) in Difco Nutrient Broth medium (Becton, Dickinson and Company, Sparks, USA). After cultivation for 16 h at 37°C and 180 rpm, main cultures (500 mL, grown in 1 L standard flasks covered with aluminum foil) were inoculated to a starting optical density (OD_600_) of 0.02 and further incubated for 4, 8, 12, 16, 20, and 24 h, respectively (37°C, 180 rpm).

### Isolation of bacterial membrane vesicles

Bacterial cultures were first centrifuged at 15,000 × *g* for 10 min at 4°C (Heraeus Megafuge 16R, Thermo Scientific, Waltham, USA). Cell-free supernatants were filtered through 0.22 µm PES bottle top filters (Fisher Brand, New Hampshire, USA) and concentrated 10-fold by tangential flow filtration (VivaFlow 200, 100 kDa cutoff, polyether sulfone, Sartorius, Göttingen, Germany). The concentrate was then ultracentrifuged for 2 h at 150,000 × *g* and 4°C in a Beckman Optima LE-80K (Beckman Coulter, Brea, USA). The supernatant was discarded, and the pellet was washed twice in 1 mL ultrapure water. Afterward, the BMV pellet was resuspended in 1 mL ultrapure water and stored at 4°C for a maximum of 1 week. Stability was confirmed for up to 2  weeks by DLS and electrophoretic light scattering (ELS), showing no changes in size, polydispersity index (PDI), or zeta potential.

### Protein, lipid, and LPS quantification

Protein amount was determined using the Pierce BCA Protein Assay Kit (Thermo Scientific, Waltham, USA) per manufacturer’s instructions. BMV isolates were either untreated or supplemented with 2% (wt/wt) SDS (Merck KGaA, Darmstadt, Germany). Lipid quantification was performed via the sulfo-phospho-vanillin (SPV) assay as previously described using 1,2-dioleoyl-sn-glycero-3-phosphocholine-based (Lipoid GmbH, Ludwigshafen, Germany) liposomes as a calibration reference (30). Lipopolysaccharide (LPS) content was measured with the Pierce Chromogenic Endotoxin Quant Kit (Thermo Scientific, Waltham, USA), using 80% of the recommended volumes while maintaining the same ratios. Samples were previously diluted to 10 µg/mL protein.

### Dynamic light scattering and electrophoretic light scattering

The intensity-dependent average hydrodynamic diameter, PDI, and zeta potential were determined using a Zetasizer NanoZS system (Malvern Pananalytical, Malvern, USA). Samples yielded directly after BMV isolation were diluted in a ratio of 1:20 in ultrapure water and measured at 25°C in polystyrene cuvettes.

### Atomic force microscopy

Muscovite mica (12 mm diameter, 0.15-0.21 mm thickness, Electron Microscopy Sciences, Hatfield, USA) was mounted on silanized microscopy slides (75 × 25 × 1 mm) and rinsed with 96% ethanol. Mica was freshly cleaved by rapidly removing a tape strip from the surface. Samples were adjusted to 0.1 µg/mL protein amount and applied in 20 µL drops on the mica surface. After air drying, the slides were stored in a desiccator overnight. All preparation steps were performed under a laminar airflow bench to exclude contaminant deposition. AFM imaging was performed with a JPK Nanowizard III (Bruker Corporation, Billerica, USA) using ACTA-50 tips (tip radius <10 nm, spring constant 37 N/m, frequency 300 kHz, 125 µm length, 30 µm width, 4 µm thickness, Al coating, APPNano, Mountain View, USA) over a 2 × 2 µm scanning area (1,024 × 1,024 resolution, 0.7 Hz line rate). Height images were processed in Gwyddion V. 2.65 (Czech Metrology Institute, among others, Jihlava, Czech Republic) with polynomial background removal and row alignment. Vesicles were detected using Otsu’s automatic particle detection algorithm; artificial diameter and subsequent total volume were determined by Laplace interpolation. Total volume was further divided by the number of identified vesicles and inserted into equation 1, with *V_L_* being the estimated volume and *d* the estimated diameter:


(1) $$d=2\cdot \sqrt[{3}]{\frac{3\cdot V_{L}}{4\cdot \pi }}.$$

### Raman spectroscopy

BMV isolates were adjusted to 10 µg/mL protein content with ultrapure water, spotted onto calcium fluoride (CaF_2_) slides (Korth Kristalle GmbH, Altenholz, Germany) in 10 µL drops and subsequently air dried. Raman spectra were acquired by line scans in the border region of dried droplets using a WITec alpha 300R + microscope (WITec GmbH, Ulm, Germany) equipped with a 50× objective (NA 0.8) and a 532 nm laser (laser power set to 10 mW in front of the objective, Carl Zeiss Microscopy GmbH, Jena, Germany). Ten accumulations (1 s integration time) ensured adequate signal intensities. Spectra were pre-processed in the Project Four software (WiTec GmbH, Ulm, Germany) by applying background subtraction and cosmic ray removal, followed by further analysis in Matlab R2022b (Mathworks, Natick, USA) using self-written scripts. All spectra were normalized to the C-H stretching peak at 2,933 cm^−1^, smoothed (Savitzky-Golay filter), and cropped to 400–1,800 cm^−1^ and 2,800–3,200 cm^−1^. Two-dimensional correlation spectroscopy (2D-COS) calculations followed published protocols (31). Raman wavenumber assignments can be found in Table 1.

**TABLE 1:** Raman wavenumber assignments

Wavenumber (cm^−1^)	Assignment	Reference
748	Tyrosine	(32)
785	Cytosine, uracil, thymine ring breathe	(28)
1,235	Amide III, β sheet	(33, 34)
1,279	Amide III, α helix	(33, 34)
1,302	Lipid C-H_2_ twisting	(35)
1,338	Carbohydrate C-C	(28)
1,645 –1,680	Amide I	(28, 36)
2,850 –2,865	Lipid C-H_2_	(28)
2,935	Aliphatic/aromatic C-H	(28, 36)

### Evaluation of cytotoxicity

THP-1 monocytes (German Collection of Microorganisms and Cell Cultures GmbH, Braunschweig, Germany) were cultivated in Roswell Park Memorial Institute 1640 medium (RPMI) supplemented with 10% fetal bovine serum, and differentiated to resting M0 macrophages as previously described (37). Cytotoxicity of BMVs was analyzed using the 3-(4,5-dimethylthiazol-2-yl)−2,5-diphenyltetrazolium bromide (MTT) assay. Briefly, THP-1 cells were seeded in a 96-well plate (40,000 cells per well) and differentiated. Resulting M0 macrophages were incubated with BMVs isolated after 4, 8, 12, 16, 20, and 24 h at concentrations ranging from 0.05, 0.5, and 5 µg/mL (protein concentration). After 24 h (37°C and 5% CO_2_), cells were washed with 1× phosphate-buffered saline (PBS) and incubated with MTT (final concentration 1 mg/mL in RPMI) for 4 h (37°C, 5% CO_2_). The supernatant was discarded, and formazan crystals were dissolved in dimethyl sulfoxide (Merck KGaA, Darmstadt, Germany). Absorbance was measured at 570 nm using the Spark multimode microplate reader (Tecan, Männerdorf, Switzerland). Cells treated with 1% Triton X-100 ([vol/vol] in RPMI) or untreated cells served as positive and negative controls, respectively, and the resulting cell viability was calculated according to equation 2 .


(2) $$Cell\;viability\;\left(\%\right)=\frac{Absorbance_{cells\;incubated\;with\;OMVs}-Absorbance_{positive\;control}}{Absorbance_{negative\;control}-Absorbance_{positive\;control}}\;\times 100.$$

### Enzyme-linked immunosorbent assays

The immunogenic potential of BMVs was evaluated via enzyme-linked immunosorbent assays (ELISA). THP-1 cells were seeded (350,000 cells per well) in a 12-well plate and differentiated. M0 macrophages were treated with BMVs (1 µg/mL protein concentration) for 6 h, and supernatants were collected. After centrifugation (10,000 × *g*, 10 min, 4°C), cell-free supernatants were stored at −80°C until further analysis. Cytokine quantification was performed using the uncoated ELISA kits for IL-1β, IL-6, and TNFα following the manufacturer’s instructions (Invitrogen, Waltham, USA).

### Statistical analysis

Statistical evaluation of data was performed with GraphPad Prism 10.2.3 (GraphPad Software, Boston, USA). An ordinary one-way ANOVA coupled with Tukey’s multiple comparisons test was applied to neighboring data to test for statistical significance, highlighting changes in small time intervals. For the ELISA results, comparisons between all data points were exceptionally performed to underscore holistic trends in cytokine results. The number of replicates performed per experiment is displayed as *N* for biological and *n* for technical replicates in the respective figure legends. Results are shown as mean values, and error bars indicate the standard deviation. Levels of statistical significance are displayed as ns (not significant), *(*P* < 0.05), **(*P* < 0.01), *** (*P* < 0.001), or **** (*P* < 0.0001). 

## RESULTS

### Isolation of bacterial membrane vesicles

**Fig 1:**
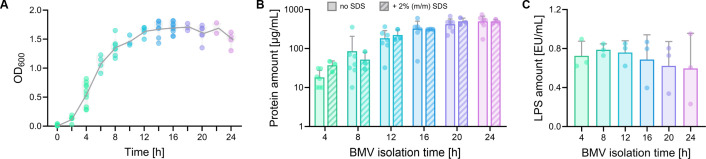
(**A**) Evaluation of *P. aeruginosa* bacterial growth by OD_600_ measurements from 0 to 24 h. The depicted line follows mean values for each time point (*N* = 5, *n* = 1). (**B**) Protein quantification of BMV samples for the isolation time points 4, 8, 12, 16, 20, and 24 h. Empty bars display protein levels in untreated samples, whereas hatched bars show concentrations in samples pre-treated with 2% (wt/wt) SDS (no SDS: *N* = 6, with SDS: *N* = 3, both *n* = 3). (**C**) LPS quantification of BMV samples for the isolation time points 4, 8, 12, 16, 20, and 24 h (*N* = 3, *n* = 3). Error bars in all graphs indicate SD. Statistical analysis of** B and C** in Table S1.

*P. aeruginosa* was grown in nutrient broth medium over 24 h with BMVs harvested every 4 h, and bacterial growth was tracked by measuring the optical density at 600 nm (OD_600_) in 2 h intervals (Fig. 1A). After a short lag phase, which lasted approximately 2 h, exponential growth was observed until 12 h of total incubation time, whereafter the OD_600_ remained consistent, indicating the stationary phase. BMV yields were quantified by determining protein amounts using a bicinchoninic acid (BCA) assay (Fig. 1B). Following the logarithmic characteristics of the growth curve, protein concentrations in BMV isolates increased exponentially. Although adding 2% (wt/wt) SDS was reported to restrain interferences between non-protein compounds and the assay, no differences were observed in this study (38). Furthermore, LPS content was assessed, revealing a slight decrease in the LPS amount over the course of bacterial growth (Fig. 1C).

### Physicochemical characterization

**Fig 2:**
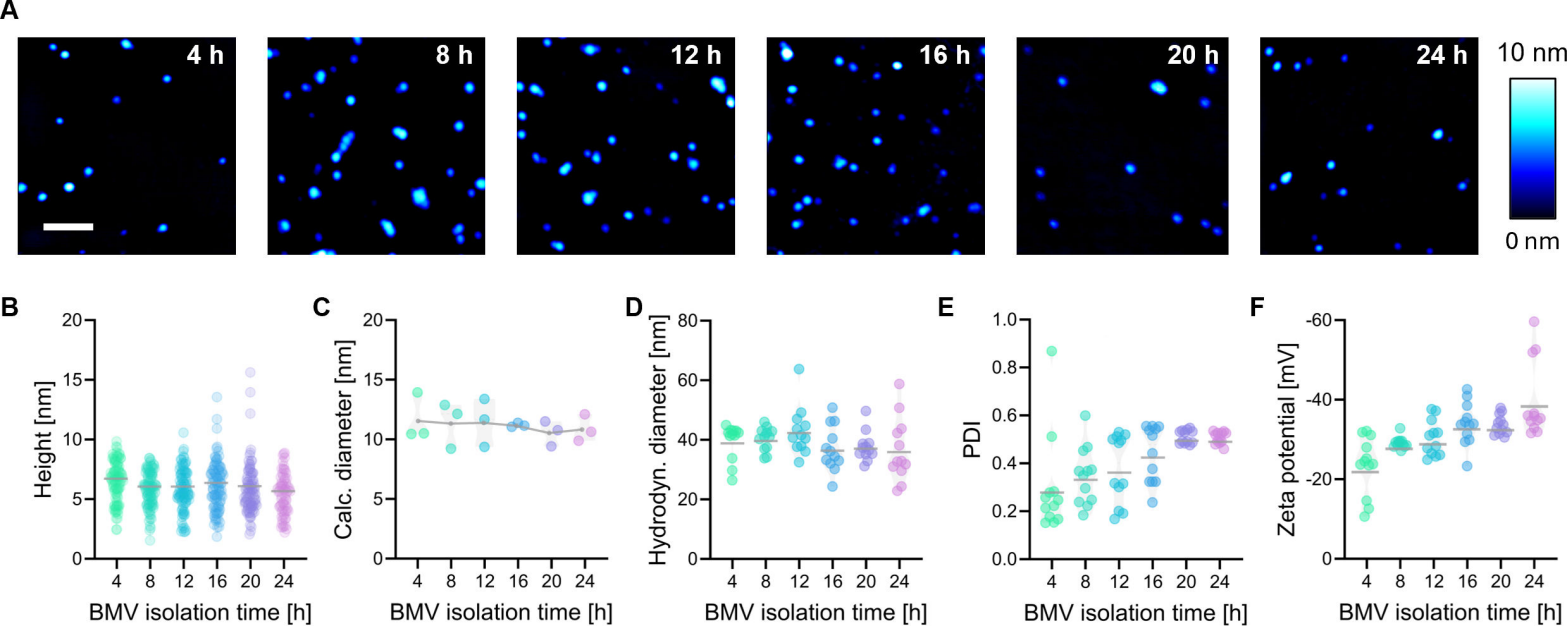
(**A**) Representative AFM height images of dehydrated BMVs from all isolation time points (4, 8, 12, 16, 20, and 24 h). Color bar on the right indicates height-color-correlation, and scale bar indicates 200 nm (total image size 1 × 1 µm). (**B**) Height values obtained from AFM images (*N* = 3, *n* = 30). (**C**) Calculated theoretical diameter from estimated volumes covered by dehydrated BMVs (*N* = 3, *n* = 30). (**D**) Hydrodynamic diameter determined by DLS (*N* = 4, *n* = 3). (**E**) PDI determined by DLS. Gray lines represent the mean values at the respective isolation times (*N* = 4, *n* = 3). (**F**) Zeta potential determined by ELS (*N* = 4, *n* = 3). Gray lines indicate the mean values at the respective isolation times. Statistical analysis of **B–F** in Table S2.

AFM was employed to evaluate the dimensional properties of the isolated BMVs. Exemplary records are shown in Fig. 2A for all isolation times. AFM confirmed the presence of spherical objects and the absence of any unwanted bacterial remains such as pili or flagella. The average height of the dehydrated vesicles is depicted in Fig. 2B. Height values were consistent at around 6 nm for all time points. Based on these measurements, diameter values were calculated from the volume covered by the deposition area, assuming the vesicles to be perfect spheres before deposition (Fig. 2C). For all isolation times, comparable artificial diameters were obtained, ranging from 9 to 14 nm. These analyses were complemented by dynamic light scattering, which determined hydrodynamic diameters between 30 and 40 nm for all BMV isolates (Fig. 2D). Using DLS measurements, the increased polydispersity of BMV populations was detected in dependence on the bacterial growth phase, with PDI values rising from 0.2 to 0.5. However, it could be noted that fluctuations in recorded PDIs decreased with time (Fig. 2E). Assessing the zeta potential with ELS revealed that surface charges of BMVs increased throughout bacterial growth, starting at −22 mV and reaching values up to approximately -40 mV (Fig. 2F).

### Raman data acquisition

**Fig 3:**
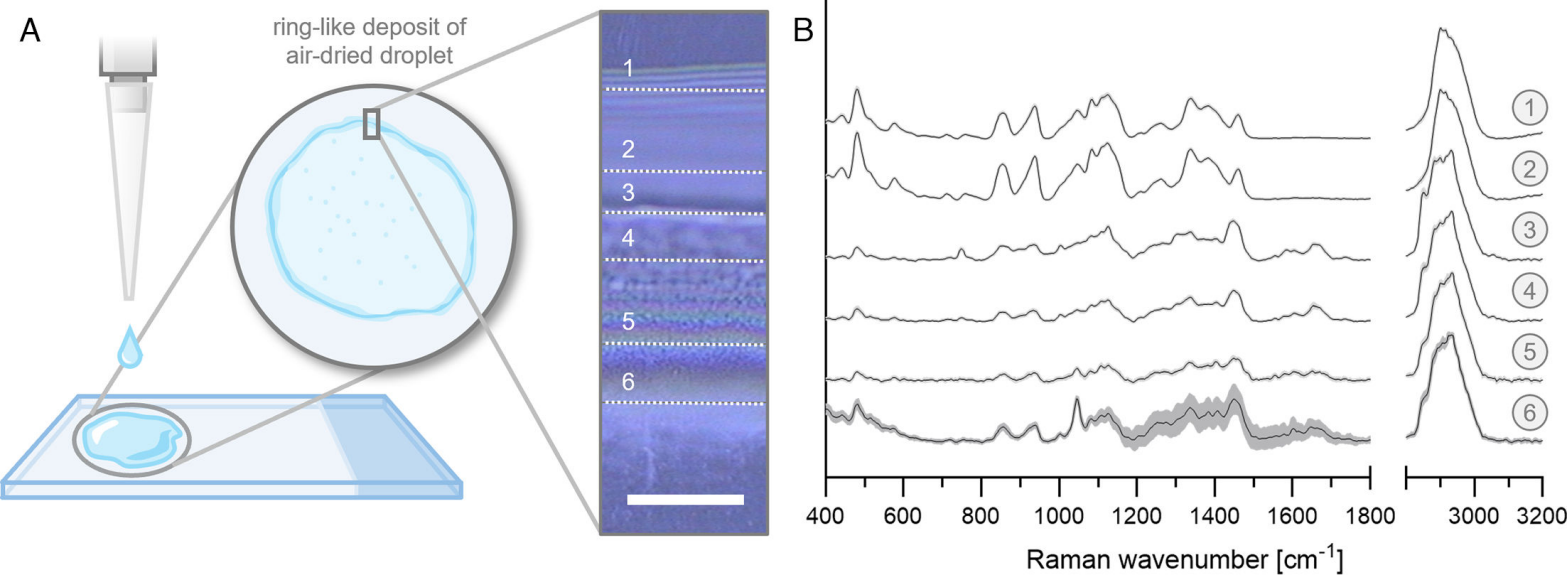
(**A**) Exemplary border analysis of the coffee ring formed out of dried-down BMV suspension (isolated after 20 h of bacterial growth). Schematic illustration of deposition with a bright field image showing the border of the dried droplet (scale bar 20 µm, outer to inner border from top to bottom), wherein six representative lines for Raman analysis are marked. (**B**) Mean Raman spectra from the six line scans are displayed with their respective SD and numeration corresponding to the respective lines in the bright field image (*n* = 10).

For Raman spectroscopy, all BMV suspensions were dried on CaF_2_ slides, creating a deposition of BMV material according to the coffee ring effect (Fig. 3A). A systematic analysis of the border region of the dried sample was performed to identify areas containing BMVs for the following measurements. Raman spectra were recorded in parallel lines at increasing distances from the border. The selected six representative lines were distributed over the border region based on visible alterations, allowing reproducible measurements of subsequent samples. As demonstrated in Fig. 3B, the respective border regions not only appeared different in bright field images but also exhibited distinct Raman spectra, indicating variations in their chemical composition. The mean spectra of line scans 1 and 2 shared identical spectral features, although both lacked protein-associated peaks, such as the prominent amide I band at 1,650–1,680 cm^−1^, suggesting the absence of BMV structures in these areas. Moving to more inner border districts, spectra from lines 3–5 indicated BMV deposition, as they contained a significant amide I signal; however, they exhibited variations in diverse peaks, such as the lipid C-H peak at 2,850 cm^−1^ and the tyrosine peak at 748 cm^−1^. Spectra collected on line six at the inner border edge gave weak signals, resulting in a comparably high SD and indicating low sample density. Among all detected peaks, some match with those outstanding in lines 1 and 2 (e.g., at 854, 939, and 1,046 cm^−1^) without appearing in lines 3–5.

Based on these results, all further scans were performed as line scans in the exact middle of the visually highlighted structured area (between lines 3 and 5), ensuring comparability and uniformity for all data.

### Chemical profiling by Raman spectroscopy

**Fig 4:**
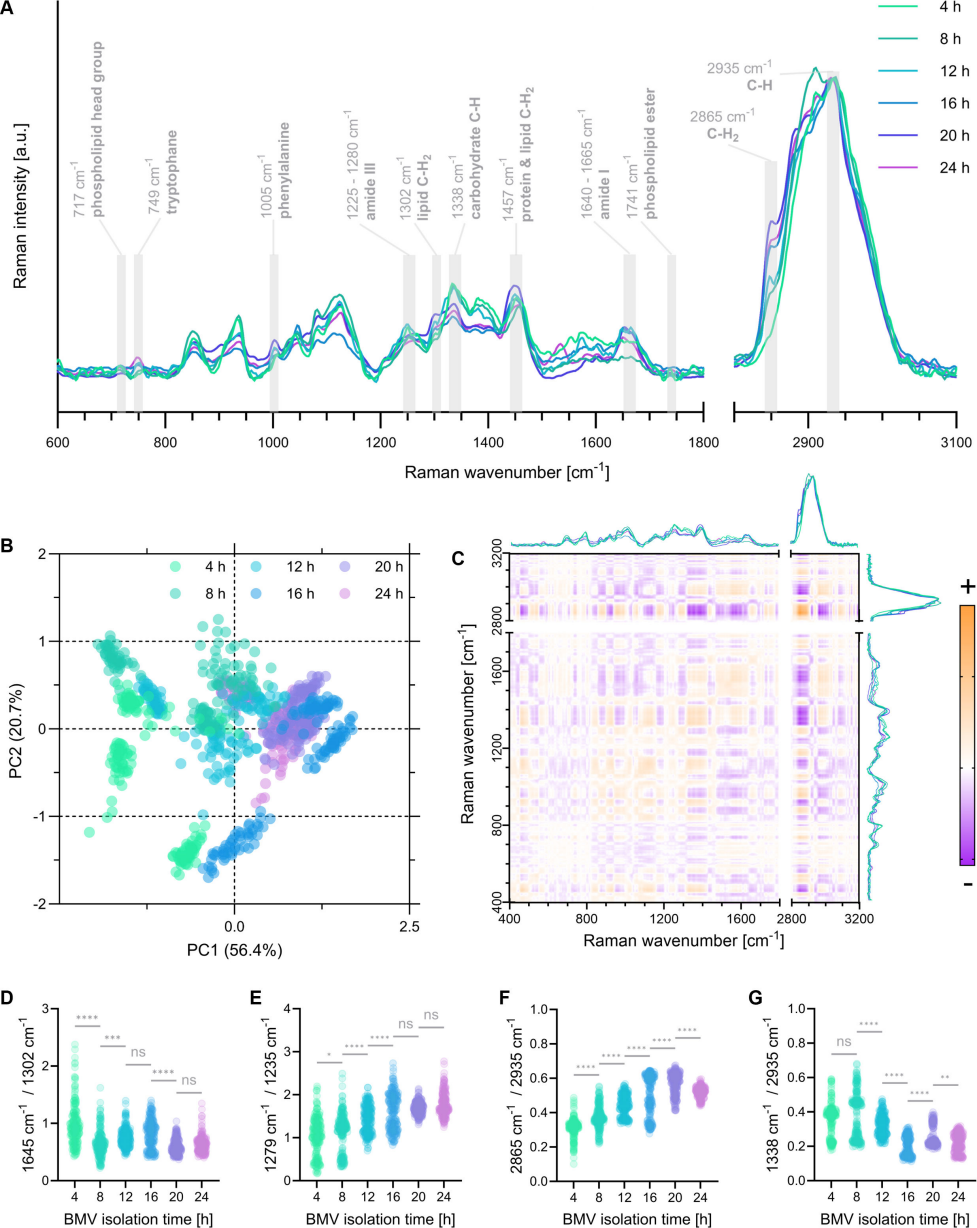
(**A**) Mean Raman spectra of all BMV isolates normalized to the C-H peak at 2,935 cm^−1^. Prominent Raman bands are annotated and marked in gray (*N* = 4, *n* = 50). (**B**) PCA of all BMV spectra (*N* = 3, *n* = 50). (**C**) 2D-COS analysis of mean spectra of all BMV isolates. Orange: positive correlation; purple: negative correlation. (**D–G**) Peak ratio analysis of distinct spectral features of BMV isolate spectra from 4, 8, 12, 16, 20, and 24 h, respectively. Statistical analysis: one-way ANOVA and Tukey’s post-hoc test (*N* = 4, *n* = 50, full statistical analysis in Table S3). Statistical significance is indicated by *(*P* < 0.05), **(*P* < 0.01), *** (*P* < 0.001), or **** (*P* < 0.0001), and ns (not significant). (**D**) Protein-to-lipid ratio (amide I band at 1,645 cm^−1^, lipid C-H_2_ twisting at 1,302 cm^−1^). (**E**) Protein secondary structures ratio (amide III band; α-helix at 1,279 cm^−1^, β-sheet at 1,235 cm^−1^). (**F**) Saturated lipid peak ratio (C-H_2_ bonds at 2,865 cm^−1^, C-H bonds at 2,935 cm^−1^). (**G**) Carbohydrate peak ratio (carbohydrate C-C bonds at 1,338 cm^−1^, C-H bonds at 2,935 cm^−1^).

Raman spectra of all BMV isolates were collected, and the resulting mean spectra are depicted in Fig. 4A, showing annotations for all relevant peaks. By performing a principal component analysis (PCA), the clustering of the six different BMV isolations was illustrated (Fig. 4B). The distribution reveals variations, especially at earlier isolation time points (4 and 8 h), with the most significant separation along PC1, accounting for 56.4% of total variance. Apparent clustering was observed at especially early time points, whereas overlapping increased for later sampling points (16 and 24 h). Group-internal deviations decreased with longer growth duration; however, distinguishing between later isolates became increasingly more complex.

An overview of all dynamic changes in relative signal intensities between isolations was visualized using 2D-COS (Fig. 4C). The symmetrical 2D-COS plot revealed all peak relationships by color-mapping positive and negative correlations of spectral information over the impact of isolation times. For example, examining the 2,865 cm^−1^ (lipid C-H_2_ peak) from both the x- and y-plotted mean spectra reveals an intense orange blot, indicating a positive correlation. Hereby, an increase in the BMV isolation time of the regarded peak on one axis was accompanied by an increase in intensity for the desired peak on the other axis. A strong, exemplary negative correlation (purple blot) could be observed by comparing the lipid C-H_2_ intensity at 2,865 cm^−1^ to the carbohydrate C-C signal at 1,338 cm^−1^, where the intensity trends of both peaks are inversely correlated. The 2D visualization of time-dependent changes in the spectroscopic data set allowed the identification of relevant Raman peak pairs for subsequent peak ratio analysis. Calculating peak ratios (Fig. 4D through G) enabled more precise insights into distinct spectral features and their semi-quantitative assessment throughout bacterial growth. Figure 4D displayed the protein-to-lipid ratio by analyzing the relation of amide I intensity at 1,645 cm^−1^ to lipid-assigned C-H_2_ twisting at 1,302 cm^−1^ and revealed highly significant differences in protein portion based on the lipid content between isolations at 4 and 8 h, 8 and 12 h, as well as 16 and 20 h. Figure 4E illustrates the detected shifts in predominant protein secondary structure by comparing two points in the amide III bands corresponding to α-helices at 1,279 cm^−1^ and to β-sheets at 1,235 cm^−1^. According to these findings, the proportion of α-helix structures in total protein structures increased with later BMV isolates, resulting in significant differences, especially between isolations up to 16 h. Furthermore, an increase in lipid saturation (Fig. 4F) in vesicles throughout bacterial growth was observed when relating the C-H_2_ signal at 2,865 cm^−1^ to the C-H signal at 2,935 cm^−1^, revealing highly significant differences between all isolation points. Another peak ratio calculated to address the carbohydrate content in BMVs showed a decrease in sugar structures (carbohydrate C-C bonds, 1,338 cm^−1^) with ongoing cultivation durations when normalized to the C-H peak at 2,935 cm^−1^ (Fig. 4G), which were especially prominent in later BMV isolates.

### *In vitro* immunogenicity

The immunogenic potential of BMVs from different isolation time points was analyzed by treating resting macrophages (M0) with 1 µg/mL (protein concentration) BMVs for 6 h. The secretion of the pro-inflammatory cytokines IL-1β, IL-6, and TNF-α was then assessed using ELISA. Beforehand, the cytotoxicity of BMVs was evaluated by an MTT assay, revealing no significant toxic effects on M0 macrophages at the chosen concentration (see Fig. S1). BMVs from the different isolation time points influenced the respective cytokine releases diversely, depending on the investigated cytokine. IL-1β levels consistently decreased when M0 macrophages were treated with later BMV isolates, reaching cytokine concentrations that do not differ significantly from the negative control in BMVs from 20 to 24 h. The IL-1β release of nearly 45 pg/mL for BMVs isolated at 4 h exceeded the immune response observed in the positive control by almost 80%. In contrast, BMVs from every isolation triggered comparable IL-6 secretion, inducing the release of over 100 pg/mL. Regarding the release of TNF-α, all isolations induced a significant immune response, and a slight increase was detected when comparing the 4 h and the 24 h isolate, where TNFα levels rose from approximately 1,800 pg/mL to 2,300 pg/mL, resembling an increase of about 28%.

## DISCUSSION

Understanding the dynamics of membrane vesicle release throughout the lifecycle of *P. aeruginosa* is crucial to deciphering its adaptive strategies. Here, we employed a combined approach utilizing established biochemical assays and, for the first time in this context, Raman spectroscopy to elucidate vesicle characteristics at defined time points. By tracking the optical density at 600 nm throughout 24 h, the temporal sequence of different growth phases was recorded (Fig. 1A). The exponential character of the growth curve corresponded to the trend in quantified protein concentrations in all collected vesicle isolates (Fig. 1B), revealing a correlation of total bacteria mass to isolated bacterial vesicle amounts. In addition to protein quantification, lipopolysaccharide contents were evaluated, showing an exponential trend with a slight non-significant decrease at later isolation times (Fig. 1C).

After BMV isolation, the purity of isolates was assessed using DLS and AFM analysis. Both measurements highlighted the absence of any larger biological debris, such as bacterial pili or flagella (Fig. 2). The average hydrodynamic diameter was approximately 40 nm, remaining almost consistent over the course of growth. Regarding the polydispersity index, variations in BMV size increase with later isolation times, revealing a broad size distribution for BMVs. According to the literature, bacterial vesicles from *P. aeruginosa* range in hydrodynamic diameter from 25 to 200 nm, in compliance with the measured sizes in this study (39–41). AFM measurements exhibited height values of BMVs of around 6 nm, which were drastically lower than previously reported values, stating heights for OMVs from *P. aeruginosa* of 10–30 nm (41). However, these height values were detected when the vesicles were retained in a hydrated state, whereas in this study, the vesicles were dried after deposition. The hereby acquired values can be attributed to twice the thickness of a lipid bilayer (approximately 4 nm), further flattened by deformation processes during drying and storage (42, 43). To obtain a more meaningful indicator of the size changes across isolation time points from AFM data, an artificial diameter was calculated from the volume covered by the deposition area, assuming the vesicles to be perfect spheres (Fig. 2C). This attempt has previously been proven to comply with particle sizes acquired by cryogenic electron microscopy, although particles need to be hydrated for best accuracy (43). In this case, the calculated diameters rather provide a means for relative comparison rather than absolute determination. Regardless of the employed method, no significant changes in BMV size were observed between the different isolation time points.

For Raman spectroscopic evaluations, BMV isolates were drop casted and fully dried before analysis. During the drying process, particle-like structures accumulate in the border region of dried droplets, a phenomenon known as the coffee ring effect (44, 45). Raman spectra were therefore recorded in this region, allowing for the best possible comparison between different samples. Analysis of the border region, however, revealed notable differences in the acquired Raman spectra depending on the position of spectra acquisition (Fig. 3). In the outer regions 1 and 2, similar spectra were recorded that can be assigned to carbohydrate structures consisting of 1,4-linked α-glucose polymers like glycogen or amylose (46, 47). Although the secretion of such polysaccharides from *P. aeruginosa* has not yet been reported, extracellular glycogen accumulation during growth in rich medium conditions was already observed for its direct relative, *P. fluorescens* (48). A deposition overlap of BMV structures with such carbohydrates cannot be excluded; nonetheless, spectra recorded across the border area revealed distinct signals of both entities in the analyzed regions. Summarizing these findings, the center area of the border is the most suitable for collecting comparable spectra of deposited bacterial vesicles, as it offers the densest deposition layer, preventing susceptibility to variations in composition. Still, exact spatial discrimination of coffee ring border entities remains a major challenge with potential implications for the gathered results. Further purification of isolated BMVs could also reduce confounding interferences, as spectral signatures not associated with bacterial vesicles were detected.

Regarding the Raman spectra of all BMV isolates (Fig. 4A), the absence of nucleic acid-associated peaks, such as cytosine, uracil, and thymine ring breathing modes at 785 cm⁻¹, indicates that DNA and RNA were not present in the analyzed vesicles (28). This suggests that lysis-derived vesicles, typically carriers of nucleic acids, are present only in minimal amounts, and the majority of isolated vesicles are outer membrane vesicles, which lack genetic material. By monitoring the presence of nucleic acids, Raman spectroscopy can be employed to discriminate between lytic vesicles and OMVs as an alternative to PAGE analysis for confirming vesicle populations after fractionation (49). PCA was applied to assess the consistency and variance across biological replicates. Early isolation time points exhibited higher variability (Fig. 4B), likely due to lower vesicle yields during the early growth phases when protein concentrations are still increasing (Fig. 1A and B). Additionally, the rapid metabolic and structural changes in bacteria during early exponential growth may cause subtle timing differences in sampling, which can disproportionately affect vesicle composition. PCA also enabled discrimination between vesicle populations from different isolation time points. Since PC1 captured the majority of variance in the data set, its loading spectra were further analyzed to identify the primary contributors to this separation (Fig. S3). The most prominent contribution was observed at 2,850 cm⁻¹, corresponding to symmetric CH_2_ stretching vibrations, a spectral marker for lipids. This indicates that lipid content is a key factor differentiating BMVs across isolation times and is a major driver of spectral variance along PC1. To further explore correlations between spectral features across the isolation timeline, symmetrical 2D-COS was employed. This method highlights dynamic relationships between Raman peaks in response to systematic changes, in this case, bacterial growth duration prior to vesicle isolation (31). Peak ratios providing crucial discriminative information about chemical composition between BMV isolates were identified. Viewing the protein-to-lipid ratio in Fig. 4D, protein content decreased relative to the lipid amounts. Unfortunately, no suitable biochemical assays are available for conducting lipid quantifications in such highly complex samples that could verify a similar trend with established assays. Both the Stewart and the SPV assays, two established and commonly applied methods for quantifying phospholipids in microorganisms or liposomes, were highly prone to harsh deviations and interfering substances, thus yielding unreliable results for bacterial membrane vesicles (the Stewart assay failed; SPV assay results are shown in Fig. S2) (50, 51). However, protein amounts are easily accessible by employing the widely accepted BCA assay, a standard method for assessing bacterial vesicle yields (Fig. 1B) (52, 53). Combining these results with Raman spectroscopy circumvents the limitations of current lipid assays and enables conclusions to be drawn regarding lipid content. Diving deeper into protein analysis by evaluating the amide III signals, α-helical structures were found to be more prominent compared to β-sheets with continuous bacterial growth (Fig. 4E). Typical transmembrane proteins present in bacterial vesicles, such as porins, primarily consist of β-barrel conformations in their transmembrane domain (7, 54). This reveals higher α-helix proportions combined with overall decreasing protein amounts in later vesicle isolates, potentially indicating a decrease in transmembrane protein abundance. Furthermore, viewing the saturation status of lipids in BMVs (Fig. 4F), later vesicle isolates show higher lipid saturation than earlier ones. It has been reported that the remodeling of lipid bilayer structures during bacterial growth is conducted in response to environmental stimuli (55). Thereby, increased phospholipid integration into the outer membrane, as well as hepta-acylation of lipid A in LPS molecules, modifies membrane stability and leads to higher abundances of saturated fatty acids in subsequently released BMVs (56, 57). The saturation deviations between the isolation time points could thus be attributed to such changes in the bacteria’s lipid bilayer composition.

**Fig 5:**
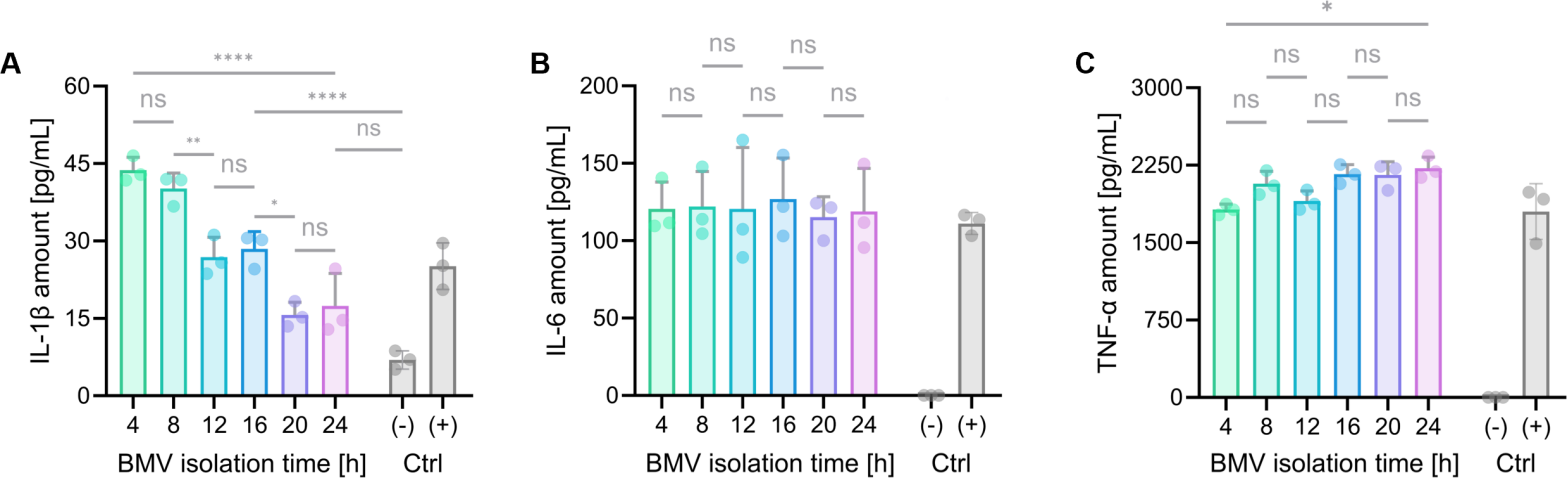
ELISA of M0 macrophages treated with 1 µg/mL of the respective BMV isolate (4, 8, 12, 16, 20, and 24 h). Investigated cytokines are IL-1β (**A**), IL-6 (**B**), and TNF-α (**C**). Negative (Ctrl-, vehicle) and positive control (Ctrl+, 50 ng/mL LPS) are depicted in gray bars. Error bars indicate SD. Statistical analysis: one-way ANOVA and Tukey’s post-hoc test (*N* = 3, *n* = 3, full statistical analysis in Table S4). Statistical significance is indicated for selected comparisons by *(*P* < 0.05), **(*P* < 0.01), or **** (*P* < 0.0001), and ns (not significant).

Lower carbohydrate levels were detected in samples from later isolation times (Fig. 4G), corresponding to the previous LPS quantification (Fig. 1C). As LPS molecules are typically classified as pro-inflammatory stimuli, it is notable that their decreasing levels did not uniformly lead to declining secretions of the analyzed classical pro-inflammatory cytokines, TNF-α, IL-1β, and IL-6, by resting macrophages treated with the six BMV isolates (see Fig. 5) (58–60). *P. aeruginosa* OMVs are reported to activate the Toll-like receptor 4 pathway in eukaryotic cells, causing secretion of pro-inflammatory cytokines such as IL-1β and IL-6, which was also observed in this study (61). Interestingly, there is no apparent trend in triggering a general immune response; instead, each observed cytokine exhibits a distinct course of release. Considering the release kinetics of the cytokines, TNF-α is a fast-acting mediator that is immediately formed when an immune response is triggered. In contrast, IL-1β levels rise more slowly and are sustained for a more extended period, typically around 24 h. IL-6 release lies midway between the two (62). ELISA data thereby revealed a gradual decrease in secreted IL-1β after treatment of resting macrophages with BMVs collected at later time points, whereas IL-6 and TNF-α secretion remained consistently high for all samples. The results could indicate a slower or delayed immune response in BMVs sampled at later time points, potentially due to different compositions of the vesicles triggering distinct inflammatory pathways. As LPS levels do not always directly correlate with the immune response, other microbe-associated molecular patterns (e.g., peptidoglycan and flagellin) or host damage-associated molecular patterns (e.g., HMGB1 and ATP) present in the BMVs may also contribute, as they can activate pattern recognition receptors and trigger cytokine production (63). Although further analyses are required to reveal the exact underlying mechanisms, these findings further emphasize the highly variable characteristics of BMVs collected after different bacterial growth times.

In our study, employing the applied analytical toolset enabled precise and specific discrimination of bacterial membrane vesicles secreted during the different growth stages of *P. aeruginosa*. It is important to note that the BMVs characterized in this study were derived from planktonic cultures under standard growth conditions, without stressors or exogenous inducers. The compositional changes observed within the first 24  h likely reflect bacterial adaptation to the environment, including accumulation of cell lysis products and nutrient depletion, as well as the onset of early biofilm formation. As reported in the literature and observed here, extended growth can lead to the formation of small bacterial aggregates (64, 65). While the amount of aggregation in our cultures is limited and unlikely to substantially affect the BMVs, it should be considered when interpreting changes in BMV composition over time. In addition to increasing lipid levels, diminished carbohydrate and protein amounts were detected, while α-helical protein structures became simultaneously more prominent. Additionally, immune responses observed in resting macrophages triggered by BMVs differ significantly between vesicles harvested at various growth stages. These findings highlight the importance of uniform and standardized isolation procedures when working with bacterial vesicles. As even minor deviations in the isolation time point were revealed to have a highly significant impact on the vesicles’ attributes, experimental statements might be dramatically influenced. Raman spectroscopy has been demonstrated to be a readily accessible technique that offers valuable insights into various molecular aspects of BMVs, including lipid saturation and protein conformation, which would otherwise be limited to LC-MS applications. These features qualify Raman spectroscopy as a powerful tool for the quality control of vesicular systems, with already reported applications for liposomes intended for therapeutic use or the classification of exosomes (66, 67). There are notable drawbacks, though, as the exact quantification of particular contents is hardly possible, and the direct identification of compounds, such as specific proteins, cannot be achieved. However, when complemented with physicochemical characterization in the form of DLS, ELS, and AFM, a comprehensive evaluation of bacterial vesicles can be achieved with minimal sample volume requirements. Bacterial vesicles will remain a hot scientific topic for the near future, and with growing interest, ensuring comparability by defining strict standards regarding experimental protocols and applying high-fidelity analytics will be key to bundled and targeted scientific efforts.

## SUPPLEMENTAL MATERIAL

Supplemental Figures and Tables jb.00410-25-s0001.pdf Figures S1 to S3 and Tables S1 to S4.

ASM does not own the copyrights to Supplemental Material that may be linked to, or accessed through, an article. The authors have granted ASM a non-exclusive, world-wide license to publish the Supplemental Material files. Please contact the corresponding author directly for reuse.
