# Supplementary material for: Raman spectroscopy reveals growth phase-dependent molecular differences in bacterial membrane vesicles
Source: J Bacteriol. 2025 Dec 5;208(1):e00410-25. doi: 10.1128/jb.00410-25 (PMC12826046; doi:10.1128/jb.00410-25)
Supplement: Supplemental Figures and Tables — Figures S1 to S3 and Tables S1 to S4. [file jb.00410-25-s0001.pdf]

Raman spectroscopy reveals growth phase-dependent molecular differences in bacterial membrane vesicles

Lennart Christe[1], Annika Haessler[1], Stefanie Gier[1], Bernd Schmeck[2,3,4,5,6], Nathalie Jung[1], Maike Windbergs[1]

[1] Institute of Pharmaceutical Technology, Goethe University Frankfurt, Frankfurt am Main, Germany

[2] Institute for Lung Research, Philipps University Marburg, Marburg, Germany

[3] German Center for Lung Research (DZL), Giessen, Germany,

[4] German Center for Infectious Disease Research (DZIF), Brunswick, Germany

[5] Institute for Lung Health (ILH), Justus-Liebig University, Giessen, Germany

[6] Department of Medicine, Pulmonary and Critical Care Medicine, University Hospital Giessen and Marburg, Philipps University Marburg, Marburg, Germany

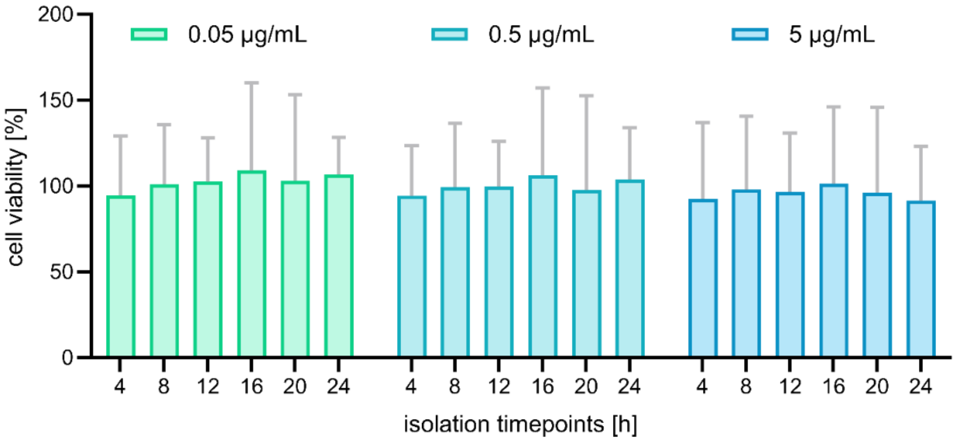

Fig. S1: MTT assay performed on M0 macrophages with OMVs isolated after 4, 8, 12, 16, 20, and 24 h in concentrations 0.05, 0.5, and 5 µg/mL (n = 3).

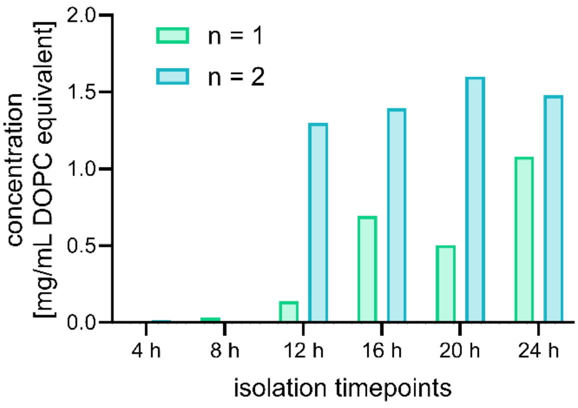

Fig. S2: Biochemical lipid assessment by sulpho-phospho vanillin (SPV) assay of OMVs isolated after 4, 8, 12, 16, 20, and 24 h (n = 2). Assay performed according to McMahon et al.

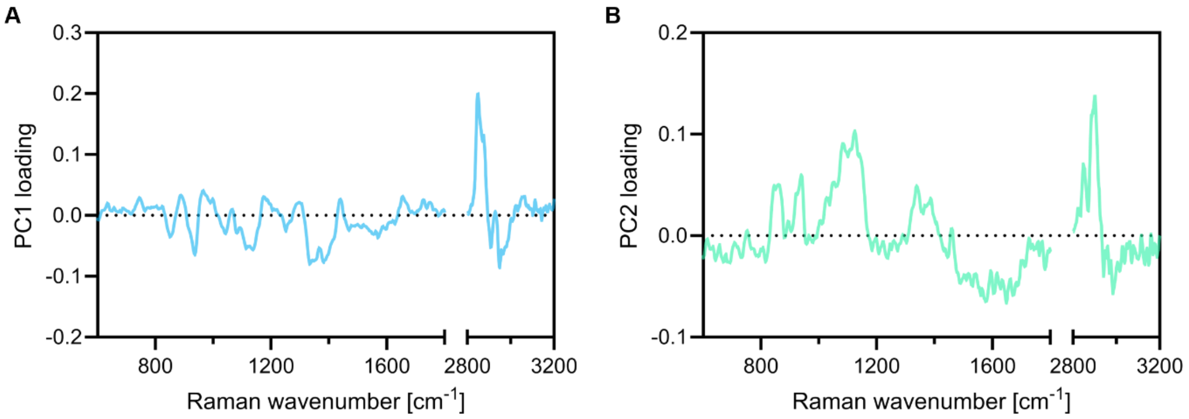

Fig. S3: Loading spectra for PC1 (**A**) and PC2 (**B**) used in the Principal Component Analysis in fig. 4B.

References:

McMahon A, Lu H, Butovich IA. The spectrophotometric sulfo-phospho-vanillin assessment of total lipids in human meibomian gland secretions. Lipids, May;48(5):513-25 (2015). doi: 10.1007/s11745-013-3755-9

Tab. S1: Statistical evaluation by one-way ANOVA and Tukey's multiple comparison test of protein and LPS quantification shown in fig. 1B and 1C.

One-way ANOVA and Tukey's multiple comparison test						
Compared groups	**Protein amounts (no SDS)**		**Protein amounts (with SDS)**		**LPS amounts**	
	Summary	p-value	Summary	p-value	Summary	p-value
ANOVA summary	****	<0.0001	****	<0.0001	ns	0.8746
4 vs. 8	ns	0.9960	ns	>0.9999	ns	0.9992
4 vs. 12	ns	0.3346	ns	0.7736	ns	>0.9999
4 vs. 16	**	0.0022	ns	0.2311	ns	>0.9999
4 vs. 20	****	<0.0001	**	0.0014	ns	0.9916
4 vs. 24	****	<0.0001	**	0.0017	ns	0.9768
8 vs. 12	ns	0.9278	ns	0.8511	ns	>0.9999
8 vs. 16	*	0.0388	ns	0.3030	ns	0.9925
8 vs. 20	***	0.0003	**	0.0023	ns	0.9371
8 vs. 24	****	<0.0001	**	0.0027	ns	0.8901
12 vs. 16	ns	0.6408	ns	0.9989	ns	0.9985
12 vs. 20	*	0.0282	ns	0.2168	ns	0.9712
12 vs. 24	***	0.0008	ns	0.2377	ns	0.9405
16 vs. 20	ns	0.9501	ns	0.7542	ns	0.9990
16 vs. 24	ns	0.3302	ns	0.7820	ns	0.9951
20 vs. 24	ns	0.9887	ns	>0.9999	ns	>0.9999

Tab. S2: Statistical evaluation by one-way ANOVA and Tukey's multiple comparison test of AFM height (fig. 2B), calculated diameter (fig. 2C), hydrodynamic diameter (fig. 2D), PDI (fig. 2E), and zeta potential (fig. 2F).

One-way ANOVA and Tukey's multiple comparison test

Compared groups	**AFM height**		**Calculated diameter**		**Hydrodyn. diameter**		**PDI**		**Zeta potential**	
	Sum.	p	Sum.	p	Sum.	p	Sum.	p	Sum.	p
ANOVA summary	**	0.0086	ns	0.9527	ns	0.2616	***	0.0001	****	<0.0001
4 vs. 8	ns	0.1295	ns	>0.9999	ns	0.9998	ns	0.8990	ns	0.1680
4 vs. 12	ns	0.1419	ns	>0.9999	ns	0.8478	ns	0.5672	ns	0.0549
4 vs. 16	ns	0.7743	ns	0.9992	ns	0.9606	ns	0.0555	***	0.0005
4 vs. 20	ns	0.1818	ns	0.9506	ns	0.9898	***	0.0008	***	0.0007
4 vs. 24	**	0.0041	ns	0.9890	ns	0.9198	**	0.0011	****	<0.0001
8 vs. 12	ns	>0.9999	ns	>0.9999	ns	0.9444	ns	0.9905	ns	0.9964
8 vs. 16	ns	0.8515	ns	>0.9999	ns	0.8790	ns	0.4453	ns	0.3329
8 vs. 20	ns	>0.9999	ns	0.9810	ns	0.9496	*	0.0224	ns	0.3914
8 vs. 24	ns	0.7570	ns	0.9977	ns	0.8054	*	0.0285	***	0.0006
12 vs. 16	ns	0.8692	ns	>0.9999	ns	0.3509	ns	0.8151	ns	0.6347
12 vs. 20	ns	>0.9999	ns	0.9745	ns	0.4824	ns	0.1039	ns	0.6999
12 vs. 24	ns	0.7354	ns	0.9963	ns	0.2691	ns	0.1263	**	0.0029
16 vs. 20	ns	0.9125	ns	0.9948	ns	>0.9999	ns	0.7323	ns	>0.9999
16 vs. 24	ns	0.1497	ns	0.9998	ns	>0.9999	ns	0.7820	ns	0.1857
20 vs. 24	ns	0.6709	ns	0.9998	ns	0.9990	ns	>0.9999	ns	0.1501

Tab. S3: Statistical evaluation by one-way ANOVA and Tukey's multiple comparison test of Raman peak ratios shown in fig. 4D-G.

One-way ANOVA and Tukey's multiple comparison test								
	**Protein-to-lipid ratio** (1645 cm^{-1} / 1302 cm^{-1})		**Protein secondary structure** (1279 cm^{-1} / 1235 cm^{-1})		**Lipid saturation** (2865 cm^{-1} / 2935 cm^{-1})		**Carbohydrate levels** (1338 cm^{-1} / 2935 cm^{-1})	
Compared groups	Sum.	p	Sum.	p	Sum.	p	Sum.	p
ANOVA summary	****	<0.0001	****	<0.0001	****	<0.0001	****	<0.0001
4 vs. 8	****	<0.0001	*	0.0269	****	<0.0001	ns	0.1713
4 vs. 12	****	<0.0001	****	<0.0001	****	<0.0001	**	0.0028
4 vs. 16	****	<0.0001	****	<0.0001	****	<0.0001	****	<0.0001
4 vs. 20	****	<0.0001	****	<0.0001	****	<0.0001	****	<0.0001
4 vs. 24	****	<0.0001	****	<0.0001	****	<0.0001	****	<0.0001
8 vs. 12	***	0.0001	****	<0.0001	****	<0.0001	****	<0.0001
8 vs. 16	****	<0.0001	****	<0.0001	****	<0.0001	****	<0.0001
8 vs. 20	ns	0.3365	****	<0.0001	****	<0.0001	****	<0.0001
8 vs. 24	ns	0.9900	****	<0.0001	****	<0.0001	****	<0.0001
12 vs. 16	ns	0.1574	****	<0.0001	****	<0.0001	****	<0.0001
12 vs. 20	****	<0.0001	****	<0.0001	****	<0.0001	****	<0.0001
12 vs. 24	****	<0.0001	****	<0.0001	****	<0.0001	****	<0.0001
16 vs. 20	****	<0.0001	ns	0.1720	****	<0.0001	****	<0.0001
16 vs. 24	****	<0.0001	****	<0.0001	*	0.0474	ns	0.0752
20 vs. 24	ns	0.7295	ns	0.0783	****	<0.0001	**	0.0013

Tab. S4: Statistical evaluation by one-way ANOVA and Tukey's multiple comparison test of ELISA data on pro-inflammatory cytokines IL-1β (fig. 5A), IL-6 (fig. 5B), and TNFα (fig. 5C).

One-way ANOVA and Tukey's multiple comparison test

Compared groups	**IL-1β**		**IL-6**		**TNF-α**	
	Summary	p-value	Summary	p-value	Summary	p-value
ANOVA summary	****	<0.0001	****	<0.0001	****	<0.0001
4 vs. 8	ns	0.9299	ns	>0.9999	ns	0.3261
4 vs. 12	***	0.0009	ns	>0.9999	ns	0.9929
4 vs. 16	**	0.0024	ns	>0.9999	ns	0.0799
4 vs. 20	****	<0.0001	ns	>0.9999	ns	0.0914
4 vs. 24	****	<0.0001	ns	>0.9999	*	0.0294
4 vs. (-)	****	<0.0001	***	0.0001	****	<0.0001
4 vs. (+)	***	0.0003	ns	0.9994	ns	>0.9999
8 vs. 12	**	0.0086	ns	>0.9999	ns	0.7510
8 vs. 16	*	0.0236	ns	>0.9999	ns	0.9861
8 vs. 20	****	<0.0001	ns	>0.9999	ns	0.9918
8 vs. 24	****	<0.0001	ns	>0.9999	ns	0.8483
8 vs. (-)	****	<0.0001	***	0.0001	****	<0.0001
8 vs. (+)	**	0.0028	ns	0.9985	ns	0.2445
12 vs. 16	ns	0.9994	ns	>0.9999	ns	0.2850
12 vs. 20	*	0.0325	ns	>0.9999	ns	0.3176
12 vs. 24	ns	0.0934	ns	>0.9999	ns	0.1199
12 vs. (-)	***	0.0001	***	0.0001	****	<0.0001
12 vs. (+)	ns	0.9987	ns	0.9994	ns	0.9742
16 vs. 20	*	0.0119	ns	0.9978	ns	>0.9999
16 vs. 24	*	0.0358	ns	0.9998	ns	0.9992
16 vs. (-)	****	<0.0001	****	<0.0001	****	<0.0001
16 vs. (+)	ns	0.9481	ns	0.9865	ns	0.0558
20 vs. 24	ns	0.9988	ns	>0.9999	ns	0.9982
20 vs. (-)	ns	0.1438	***	0.0002	****	<0.0001
20 vs. (+)	ns	0.0953	ns	>0.9999	ns	0.0641
24 vs. (-)	ns	0.0518	***	0.0002	****	<0.0001
24 vs. (+)	ns	0.2466	ns	0.9998	*	0.0202
(-) vs. (+)	***	0.0004	***	0.0004	****	<0.0001
